# Optimising the zebrafish Cre/Lox toolbox. Codon improved iCre, new gateway tools, Cre protein and guidelines

**DOI:** 10.3389/fphys.2023.1221310

**Published:** 2023-08-02

**Authors:** Alisha Tromp, Haitao Wang, Thomas E. Hall, Bryan Mowry, Jean Giacomotto

**Affiliations:** ^1^ Queensland Brain Institute, University of Queensland, St Lucia, QLD, Australia; ^2^ Institute for Molecular Biosciences, University of Queensland, St Lucia, QLD, Australia; ^3^ Queensland Centre for Mental Health Research, Wacol, QLD, Australia; ^4^ Centre for Cellular Phenomics, School of Environment and Science, Griffith Institute for Drug Discovery, Griffith University, Brisbane, QLD, Australia

**Keywords:** Cre recombinase, conditional, transgenesis, plasmids, clones, mRNA, mosaicism

## Abstract

We recently introduced the Cre/Lox technology in our laboratory for both transient (mRNA injections) and stable/transgenic experiments. We experienced significant issues such as silencing, mosaicism, and partial recombination using both approaches. Reviewing the literature gave us the impression that these issues are common among the zebrafish community using the Cre/Lox system. While some researchers took advantage of these problems for specific applications, such as cell and lineage tracing using the Zebrabow construct, we tried here to improve the efficiency and reliability of this system by constituting and testing a new set of tools for zebrafish genetics. First, we implemented a codon-improved Cre version (iCre) designed for rodent studies to counteract some of the aforementioned problems. This eukaryotic-like iCre version was engineered to i) reduce silencing, ii) increase mRNA stability, iii) enhance translational efficiency, and iv) improve nuclear translocation. Second, we established a new set of tol2-kit compatible vectors to facilitate the generation of either iCre-mRNA or iCre-transgenes for transient and transgenic experiments, respectively. We then validated the use of this material and are providing tips for users. Interestingly, during the validation steps, we found that maternal iCRE-mRNA and/or protein deposition from female transgenics systematically led to complete/homogeneous conversion of all tested Lox-responder-transgenes, as opposed to some residual imperfect conversion when using males-drivers or mRNA injections. Considering that we did not find any evidence of Cre-protein soaking and injections in the literature as it is usually conducted with cells, we tested these approaches. While soaking of cell-permeant CRE-protein did not lead to any detectable Lox-conversion, 1ng–10 ng protein injections led to robust and homogeneous Lox-recombination, suggesting that the use of protein could be a robust option for exogenous delivery. This approach may be particularly useful to manipulate housekeeping genes involved in development, sex determination and reproduction which are difficult to investigate with traditional knockout approaches. All in all, we are providing here a new set of tools that should be useful in the field.

## Introduction

The Cre/Lox technology has fast developed to be an invaluable and routinely used genetic tool in zebrafish research. By means of this technology, one uses Cre recombinase, an enzyme originally derived from a prokaryotic source to recognise and excise/invert a pair of loxP sites flanking DNA segments (TsienNeurogenetics, 2016; Carney and Mosimann, 2018; Kim et al., 2018). Given its versatile applications, we recently introduced this technology in our lab for both transient (Cre-mRNA injection) and stable/transgenic experiments. Although this technology is straightforward and effective, we experienced significant issues such as transgene-silencing, mosaicism and partial recombination using both approaches. We reviewed the literature and found that these issues are common among the zebrafish community, with some studies actually taking benefit of this variability/heterogeneity to conduct cell and lineage tracing studies using the Zebrabrow construct and line (Pan et al., 2013; Nguyen et al., 2017; Nguyen and Currie, 2018). It has been shown in rodents that the presence of multiple Cre-Driver or Lox-Responder copies/alleles could sensitise the transgenic lines to incomplete excision, also described as Cre-mosaicism (Heffner et al., 2012; Bao et al., 2013). Given that most zebrafish transgenesis is based on tol2-dependant genomic integrations which often leads to multiple insertions, the community may benefit being careful and aware of this possible problem of partial Lox-recombination within their lines and experiments (Kawakami, 2007).

To address this problem within our research, here, we started working at extending the available Cre/Lox material for zebrafish research. Upon reviewing the literature we found that most vertebrate studies had switched to a codon-optimized Cre (improved Cre or iCre) recombinase since its first introduction in the early 2000s (Shimshek et al., 2002). In contrast, the vast majority of the zebrafish studies are still using the prokaryotic version. The iCre version was initially engineered to apply the eukaryote codon usage instead of the original prokaryote one, a genetic characteristic known to significantly affect gene expression (Gustafsson et al., 2004; Zhou et al., 2016). In addition, iCre was designed to i) lower the CpG content (with high CpG demonstrated to strongly attract methylation and silencing in zebrafish (Pang et al., 2015)) ii) eliminate putative cryptic splice sites, iii) add a Kozak sequence, iv) alter the STOP codon (TAG to TGA) and complement the sequence with an optimised nuclear localization signal (NLS). All in all, in vertebrates, this has been demonstrated to significantly reduce silencing, increase mRNA stability, enhance translational efficiency and improve nuclear translocation (Shimshek et al., 2002; Gustafsson et al., 2004; Zhou et al., 2016).

**FIGURE 1:**
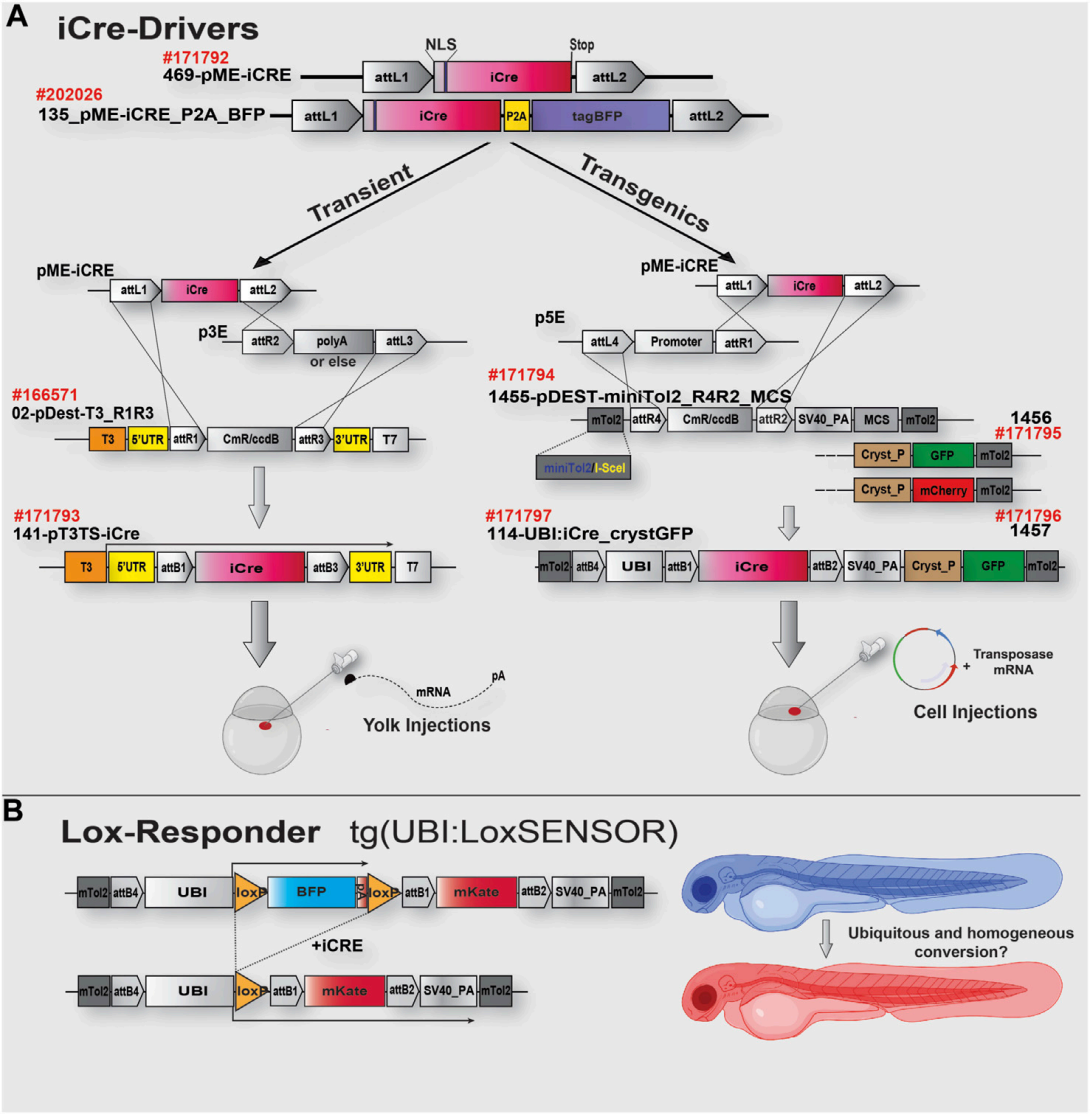
iCre toolkit and cloning strategy schematics. **(A)**. The top of the schematic shows the gateway-compatible 469-pME-iCre clone (Addgene #171792) encoding the codon-improved iCre as described in the text. This clone could be used to either generate mRNA for injection and rapid experiments (Left side, Transient) or to generate transgenes for further genomic integration and generation of iCre-Driver transgenic lines. On the left side of the upper panel (transient), optimised material for a 2-ways R1/R3 LR-rection using 02-p3T3TS_R1R3, easing success rate of the reaction and designed to improve downstream mRNA synthesis and stability. On the right side, optimised material for 2-ways R4/R2 LR reaction and transgene generation. Each pDest presents miniTol2 sequences complexed with I-SceI sequences and either a Multi Cloning Site (MCS) for rapid integration of any marker of choice or a GFP/mCherry sequence under the control of a Crystallin promoter (for Lens expression and transgenics identification). In this study, we generated the iCre-Driver construct/lines #171797, with iCre under the control of the Ubiquitin promoter (GFP Lens expression as selection marker). **(B)**. Schematic representation of the Lox-Responder sensor “UBI:LoxSENSOR” used in this study. In the absence of iCre, BFP is translated/expressed and terminated with a polyA signal. In the presence of iCre, Lox sequences are recombined, the BFP cassette is removed, triggering translation/expression of a mKate cassette. Ubiquitous expression of iCre protein, via exogenous injection or transgenic expression, should turn the animals from blue to red. Partial or mosaic conversion would be observed via the remaining BFP/Blue expression. See Table 1 for details of each plasmid.

Here, we have implemented and tested this iCre version and complemented it with a series of gateway/tol2-kit compatible plasmids, designed to ease iCre-mRNA generation and iCre-tol2-transgene constructs for transient expression and transgenesis respectively, with all material now available in Addgene (Figure 1A; Table 1). Interestingly, during the validation experiments, we found that female Cre-Drivers ubiquitously expressing Cre systematically led to complete and homogenous recombination of all our Lox-Responder transgenes, in contrast to the often imperfect recombination observed when using male Cre-Drivers. This suggested a maternal contribution and possible accumulation of iCre mRNA and/or iCre protein. Considering the absence of literature in this regard, we tested and compared the effect of mRNA and protein injections for triggering homogeneous recombination. We found that both strategies are viable with protein supplementation clearly being a viable alternative. Protein injections may also provide an attractive approach for local Lox-recombination at a later stage of the zebrafish development. All in all, we are providing here a set of new tools and approaches that should improve and facilitate Cre/Lox experiments and lines generation in the community.

**TABLE 1:** iCre/Lox toolkit components available at Addgene.

Plasmid name/Addgene ID	Addgene Code	Description/Features
469-pME-iCre	#171792	Middle Entry pME clone containing Codon-improved iCre. Main features include reduced CpG content, Kozak sequence, NLS and an altered STOP codon (TGA)
135_pME-iCRE_p2a_tagBFP	#202026	Middle Entry pME clone containing Codon-improved iCre (without stop) and fused to a P2A-BFP cassette, enabling *in vivo* tracking of iCre expression
02-p3T3TS_R1R3	#166571	2-ways R1/R3 Gateway compatible destination vector with R1/R3 sites (Designed for optimised RNA synthesis/injections). Desgined to recombine with pME and p3E constructs without the requirement for a p5E compenent. This plasmid carries β-globin-UTRs at 5′ and 3′ends as well as both T3 (sense) and T7 (antisense) promoters for RNA synthesis
141-pT3TS-iCre	#171793	Final clone ready to use synthetising iCre-mRNA using kits based on T3-promoter
1455-pDEST-miniTol2_R4R2_MCS	#171794	Custom destination clones presenting mini-Tol2 and I-SceI sequences along with a multi cloning site (MCS) for the addition of any marker of choice. Gateway compatible for a 2-ways R4R2 assembly designed to accept 1 p5E and 1 pME.
1456-pDEST-miniTol2_R4R2_Cryst-eGFP	#171795	Custom destination clones presenting mini-Tol2 and I-SceI sequences along with a marker cassette encoding eGFP under the control of ⍺-crystallin promoter. Gateway compatible for a 2-ways R4R2 assembly sites designed to accept 1 p5E and 1 pME.
1457-pDEST-miniTol2_R4R2_Cryst-mCherry	#171796	Custom destination clones presenting mini-Tol2 and I-SceI sequences along with a marker cassette encoding mCherry under the control of ⍺-crystallin promoter. Gateway compatible for a 2-ways R4R2 assembly designed to accept 1 p5E and 1 pME.
114-UBI:iCre_crystGFP	#171797	Tol2 injectable plasmid expressing iCre and crystalline eGFP under the control of the ubiquitin promoter
TAT-Cre-Recombinase	NA	Cell-permeant Cre protein including a N-terminal histidine tag, a cell penetrating peptide (TAT), and an NLS.

## Results and discussion

### Generation of a versatile gateway iCre toolbox

To try to limit Cre-mosaicism and silencing in our experiment, we established the iCre gateway-toolbox presented in Figure 1 and Table 1 (Kwan et al., 2007). First, we designed pME-vectors containing the improved Cre (iCre) version as described above as well as a fused iCre:tagBFP, named 469-pME-iCre (Addgene #171792) and 135_pME-iCRE_p2a_tagBFP (Addgene #202026). Second, designed for transient experiments, we incorporated a destination clone specifically developed for RNA synthesis, named 02-pDEST-T3TS_R1R3 (Addgene: #166571). This destination clone has been designed to incorporate elements helping mRNA’s stability and translatability, such as beta-globin 5′ and 3′UTR sequences. It has been previously demonstrated that incorporating those non-coding regions into a synthetic mRNA could lead to up to a 20-fold improvement of the associated protein expression (Fink et al., 2006; Horstick et al., 2015; Asrani et al., 2018). In addition, in contrast to traditional Tol2kit destination clones designed for three-inserts R4R3 gateway LR reaction (Kwan et al., 2007), this vector is engineered for a two-insert R1R3 assembly, allowing to recombine any middle/pME and 3′/p3E clones without the need for a 5′/p5E clone (Figure 1A). Going from three to two-insert dramatically eases and increases the success rate of the LR reaction. This backbone uses the T3 promoter and, in our hands, has proved to routinely generated a high yield of mRNA (>1–2 μg/μL per reaction). Third, designed for transgenesis, we developed several destination clones also specifically designed to ease and enhance the success of the LR reaction while limiting the overall size of the final destination transgenes, limiting potential recombination and integration problems with large constructs/cassettes. For this, we integrated mini-tol2 (Urasaki et al., 2006) sequences complexed with I-SceI (Grabher et al., 2004) sequences, an SV40 polyadenylation signal as well as a Multi Cloning Site (MCS) for users to add their markers of choice (1455-pDEST-miniTol2_R4R2_MCS, Addgene #171794). We also generated two additional constructs presenting either an eGFP or mCherry marker cassette under the control of an α-crystallin promoter -Lens expression- (Figure 1A; Table 1). All of these destination clones are designed for a two-insert gateway R4R2 LR-reaction (Figure 1A), allowing to recombine any 5′/p5E with any middle/pME (here, 469-pME-iCre) without the need to use a 3′/p3E element in the reaction mix. All these plasmids are presented in Table 1 a available at Addgene.

### Validation of iCre-mRNA induced recombination (injection)

To test the validity of our Cre/Lox toolbox, we used a “Sensor” transgenic line already available in our laboratory, named tg(UBI:LoxSENSOR), presenting a Lox-Responder transgene as illustrated in Figure 1B. In the absence of Cre, this transgene ubiquitously expressed a BFP cassette and drives strong/homogeneous blue-fluorescence. In the presence of Cre, the Lox sequences surrounding the BFP cassette recombined, thereby activating translation/expression of a mKate2 cassette and associated red-fluorescence. In short, this system enables the testing of iCre efficiency and simply observing the conversion of Blue to Red Fluorescence. Unfloxed animals or cells express blue-fluorescence only, while fully floxed animals or cells express red-fluorescence only. Similarly, partially floxed animals present simultaneous blue and red fluorescence or a mosaic-pattern.

**FIGURE 2:**
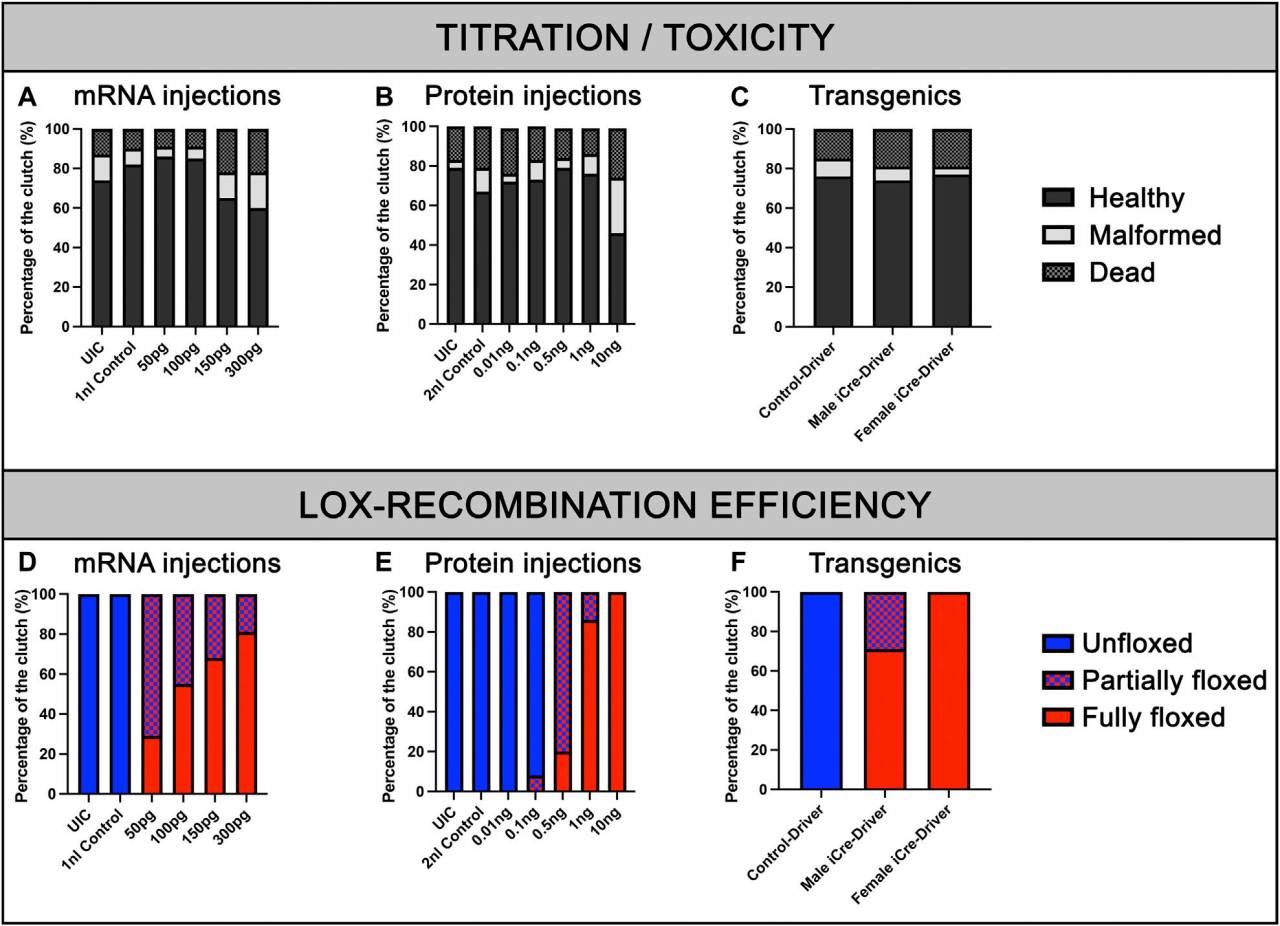
Viability of embryos and recombination efficiencies with iCre-mRNA, Cre-protein or transgenics. **(A)**, Titration of iCre-mRNA injections. Yolk injection of 50 ng/µl, 100 ng/µl, 150 ng/µl, and 300 ng/µl iCre-mRNA, 1 nl Control and uninjected controls (UIC). **(B)**, Titration of Cre protein injections. Cell injection of 0.01, 0.1, 0.5, 1, and 10 ng Cre protein, 2 nl control and uninjected control (UIC). **(C)**, F2 Clutch viability with transgenic iCre expression from F1 iCre-Driver tg(UBI:iCre). **(D)**, Quantification of Lox-recombination following iCre-mRNA injections. Lox-recombination has been assessed based on the detectable conversion/shift of BFP fluorescence to mKate fluorescence. Partial Lox-conversion is evidenced by the combined expression of both BFP and mKate fluorescence, while full Lox-conversion is evidenced by mKate expression with no detectable BFP fluorescence. Results are presented as percentages. **(E)**, Quantification of Lox-recombination following Cre-protein injections. Cre-protein injections ranging from 1 ng to 10 ng were sufficient to trigger robust and reproducible conversions. **(F)**, Quantification of Lox-recombination with transgenics. Female iCre-driver systematically triggered complete and homogeneous Lox-recombination, suggesting a robust effect of iCre mRNA and/or iCre protein maternal deposition.

**FIGURE 3:**
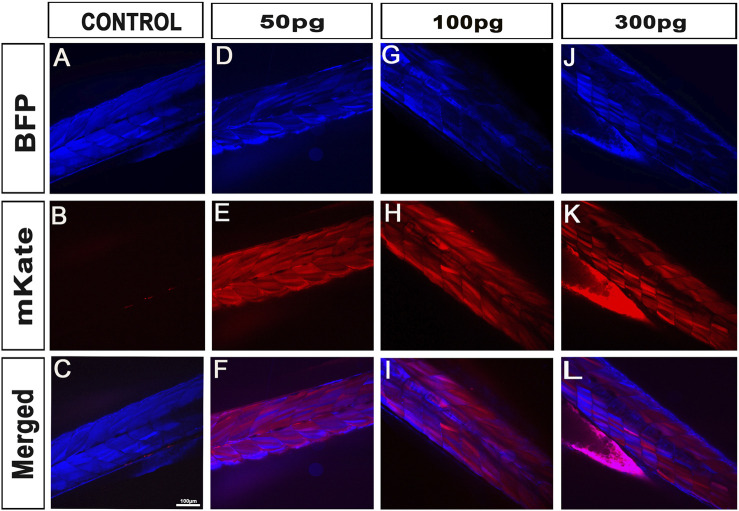
iCre-mRNA injections successfully trigger Lox-recombination but still with some visible partial conversions. **(A–C)**, Control injections demonstrating no spontaneous Lox-recombination in absence of iCre. **(D–L)**, Injection of 50pg–300 pg of iCre mRNA at one-cell stage is enough to promote Lox-recombination. However, partial conversion is still observed with the line studied. At 300 pg, most animal (81%) were however successfully fully converted with no visible blue fluorescence. Scale bar 100 µm.

To validate our kit for mRNA injections/experiments, we recombined the aforementioned 469-pME-iCre clone with 302-p3E-polyA (available from the Tol2kit (List of entry and destination vectors, 2007)) into 02-pDEST-T3_R1R3 to generate the destination clone 141-pT3TS-iCre (Figure 1A, left panel). We then synthesized stable iCre-mRNA as described below and stored the associated products at 1 μg/μL at −80°C. From this, to determine the optimal iCre-mRNA concentration to maximise loxP recombination while minimizing embryo lethality/toxicity, we injected iCre-mRNA in one-cell stage embryos at a final concentration of 50pg, 100pg, 150pg and 300 pg. We also maintained two populations of controls for the experiments, Uninjected control embryos (UIC) to evaluate the potential death rate and overall quality of each clutch. Control embryos injected with 1 nL including equal volumes of phenol red marker to quantify the effect of the microinjections *per se*, named 1 nL-control. To assess toxicity, all embryos were categorized into dead, malformed or wildtype/healthy, and data was plotted as a percentage of the whole clutch (Figure 2A). Most injections were very well tolerated, with around 74% healthy animals in the uninjected control versus 82% in the 1 nL-Control, 86% for 50pg and 85% for 100 pg. Injections of 150pg and 300 pg were also well tolerated with slightly more malformed/dead embryos and 65% and 60% of the embryos developing normally respectively. To assess loxP recombination efficiency associated with the same range of doses, we subsequently assessed change in fluorescence in the different conditions tested (Figure 2D; Figure 3). All quantifications were performed on embryos at 4 days-post-fertilisation (dpf). First, as presented in Figure 2D, only blue fluorescence was detected in the two controls. The absence of red fluorescence in 100% of the control animals demonstrates no spontaneous recombination in the absence of iCre. Second, confirming iCre-mRNA efficiency and Lox-recombination, red fluorescence was observed in all the animals injected, regardless of dose (Figure 2D). Third, as expected, not all doses led to complete homogeneous recombination. For example, while we observed that ∼81% of the embryos injected with 300 pg (1 nL injections at 300 ng/μL) present a complete lack of blue, demonstrating complete recombination of the Responder transgenes, the remaining 19% of the injected batch still expressed some detectable blue fluorescence, demonstrating partial recombination in few animals **(** Figure 2C; Figure 3 **)**. Correlated with the injected doses, complete recombination was observed in 69% of embryos injected with 150pg, in 56% injected with 100pg, and lastly only in 30% of the animals injected with 50 pg **(** Figure 2C; Figure 3 **)**. Taken together, our results demonstrate that our material and newly synthesized iCre-mRNA efficiently triggers LoxP-recombination. However, one has still to take into account the possibility of partial conversion in some of the injected animals, even in the highest doses.

### Validating iCre-induced recombination in transgenic experiments

**FIGURE 4:**
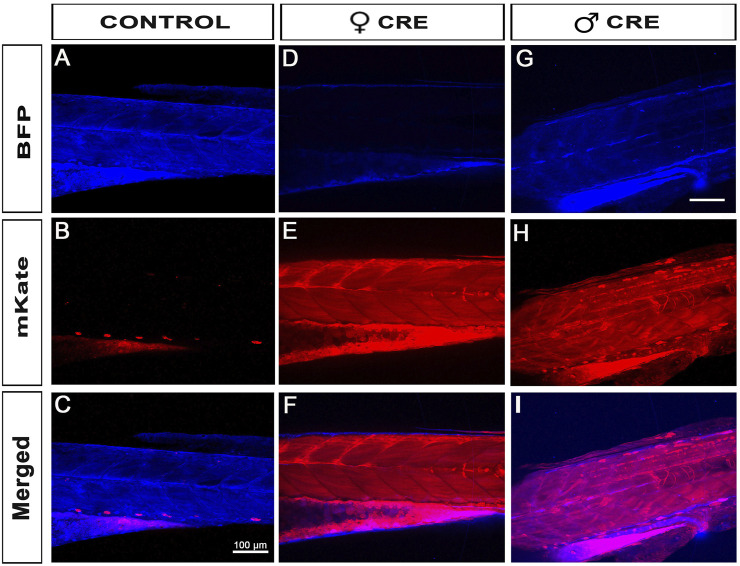
Female iCre-driver tg(UBI:iCre) triggers 100% recombination of Lox-Responder transgenes. **(A–C)**, When our Lox-Responder tg(UBI:LoxSENSOR) is crossed against a wildtype control, no Lox-recombination is observed and all analysed animals presented with homogeneous BFP/blue fluorescence. **(D–F),** When Females tg(UBI:iCre) are crossed against Males tg(UBI:LoxSENSOR), 100% all clutches observed presented with homogeneous and fully converted Lox-transgenes; *i.e.*, all animals presented Red/mKate fluorescence without any visible Blue/BFP fluorescence, evidencing complete Flox-recombination in all animals observed. **(G–I)**, When Males tg(UBI:iCre) are crossed against Females tg(UBI:LoxSENSOR), >70% of animals presented with homogeneous full Lox-recombination (Figure 2E), but a few animals remain only partially floxed, evidenced by the presence of around 30% of animals presenting both Red/mKate and Blue/BFP fluorescence. Scale bar 100 µm.

To further validate our material and iCre’s efficiency in transgenic experiments, we recombined 469-pME-iCre with p5E-UBI (5′clone containing the Ubiquitin promoter) into the optimized destination clone 1456-pDEST-miniTol2_R4R2_Cryst-eGFP (Addgene # 171795), and injected this final construct 114-UBI:iCre_crystGFP (Addgene #171797) with tol2 transposase to promote genomic integration (Figure 1; Table 1). We generated a stable F1 tg(UBI:iCre) transgenic line that ubiquitously expresses iCre as well as GFP within the lens of the eye for selection/identification of both the adults and embryos during the crossings and analysis. We then crossed this F1 tg(UBI:iCre) with tg(UBI:LoxSENSOR). Harvesting clutches of embryos from multiple crosses, we did not observe any significant toxicity as shown in Figure 2C. Confirming the validity of our constructs and iCre’s efficiency, all animals presenting green eyes did demonstrate some level of red fluorescence, evidencing proper recombination (Figure 1F). Interestingly, evaluating these transgenics’ recombination efficiencies revealed that embryos in clutches harvested from crosses between female tg(UBI:iCre) against male tg(UBI:LoxSENSOR) led to 100% efficiency in recombining loxP transgenes, *i.e.*, every single embryos had a complete absence of BFP-blue fluorescence while presenting a homogeneous mKate-red fluorescence without mosaicism (Figure 2F; Figure 4). It is important to note that 100% of the embryos/larvae from female tg(UBI:iCre) against male tg(UBI:LoxSENSOR) lines demonstrated proper recombination including those that did not inherit the iCre transgene (and therefore did not express GFP in the lens). On the other hand, male tg(UBI:iCre) against female tg(UBI:LoxSENSOR) crosses led to the generation of a significant number of larvae with residual BFP-blue fluorescence along with mKate-red expression, evidencing a subset of animals with partial conversion (Figure 2F; Figure 4). We hypothesized that the observed 100% recombination rate from the female iCre-drivers could be due to maternal deposition/contributions of both iCRE-mRNA and protein. Considering that our iCre-mRNA injections data presented above led to only partial recombination, we hypothesized that iCre protein present at an early stage could have a better efficiency for triggering homogeneous recombination. Considering we could not find any literature evidence about the use of Cre-protein, we investigated this approach by testing a commercially available Cre as discussed below. Taken together, our results validate and demonstrate the efficiency of our material for transgenesis.

### Validating induced recombination by Cre-protein injection

To investigate whether the 100% recombination rates observed using female iCre-drivers were mostly due to the presence of iCre protein, we explored our ability to reproduce this result through Cre-protein supplementation at the one-cell stage. Delivering Cre-protein into mammalian cells has been previously shown to be highly efficient and has been a widely adopted method for inducing robust Lox-recombination (Nagahara et al., 1998; Peitz et al., 2002; Nolden et al., 2006; Kang et al., 2018). Gaining inspiration from these studies, we purchased and tested a cell-permeable recombinant Cre protein (His-TAT-NLS-Cre) fused with an N terminal histidine tag, a cell-penetrating peptide (TAT), and NLS (Peitz et al., 2002; Nolden et al., 2006). Popularly used in cell culture, this soluble fusion protein suspended in cell media translocates through cell membranes and is able to initiate site-specific recombination with more than 90% efficiency (Peitz et al., 2002; Nolden et al., 2006). To test a similar delivery method, we first investigated whether Cre protein could translocate through the cell membranes of whole zebrafish embryos by immersing in a Cre-protein-enriched environment. We soaked embryos from the one-cell stage by loading the chorion of tg(UBI:LoxSENSOR) zygotes with Cre-protein (2 μg/μL to 8 μg/μL), and incubated them overnight. No impact on survival or gross development has been detected (data not shown). Unfortunately, we also did not detect any red fluorescence in any batch tested, suggesting an absence of translocation within the cells or an absence of Cre activity. We further tested if injecting Cre-protein directly into the cell would reproduce the aforementioned complete recombination of our batches. We injected one-cell staged embryos with doses ranging from 10pg to 10 ng (Figure 2E). We first analysed the overall effect of these doses on survival and development, as previously described (Figure 2E). As in the iCre-mRNA validation experiments, we included two control populations with uninjected control (UIC) and 2 nL-injected control animals. Data from our controls reported 79% and 67% healthy animals for our UIC and injected controls respectively. We then reported on toxicity effects by injecting Cre-protein and found no obvious difference except for the highest dose of 10 ng. As presented in Figure 2E, while injections of 10pg, 100pg, 500pg and 1 ng of cell-permeant Cre-protein respectively maintained a comparable ratio of healthy animals at 72%, 73%, 79% and 76%, we observed a sharp decline at 10ng, i.e., only 46% of the animals survived or presented with normal development.

**FIGURE 5:**
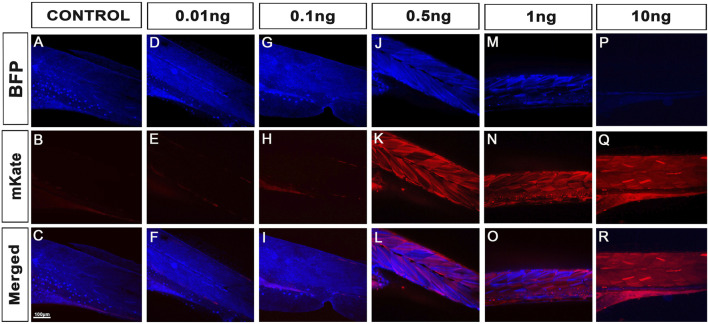
Cre protein injections successfully trigger Lox-recombination. **(A–C)**, Control injections demonstrating no spontaneous Lox-recombination in the absence of Cre protein. **(D–R)**, Injection of 10 pg to 10 ng of cell-permeant Cre protein at one-cell stage. No significant Lox-recombination is observed from 10pg to 100 pg. However, robust conversions are observed from 0.5 ng to 10ng, with 100% of the animal injected with 10 ng presenting total conversion of the Lox-transgenes (*i.e.*, only Red/mKate fluorescence was detected). Note that protein injections may also be an attractive approach for local Lox-recombination at a later stage of zebrafish development. Scale bar 100 µm.

Healthy embryos were then analysed for their fluorescence. Imaging these fish at 4dpf using confocal microscopy, we observed that both 10pg and 100 pg did not trigger any detectable/observable Lox-recombination (Figure 2E; Figure 5). Presence of red fluorescence and Lox-recombination could be detected from 500 pg but with clear mosaicism and partial conversion, 100% of the animals presenting some red fluorescence but with up to 80% with only a partial conversion and mosaic pattern. From 1ng, Lox-recombination was more successful with 86% of the animals presenting a robust recombination pattern, with a complete absence of blue-fluorescence and homogeneous red expression. Interestingly, 10 ng reproduced the previously observed full conversion obtained with maternal deposition (Figure 2E; Figure 5). However, this range of dose also seems to trigger adverse effects as presented above. Taken together, these results demonstrate that the use of Cre-protein supplementation is a viable alternative to the use of mRNA injection. It may actually be a powerful approach at later stage, to trigger local Lox-recombination using site-specific local injections or delivery of lipid nanoparticles.

## Discussion and conclusion

Here, we expanded the zebrafish Cre/Lox toolbox by incorporating codon-improved iCre and optimised gateway-compatible clones for both transient and transgenic experiments. All plasmids and tools developed here are now available at Addgene (Table 1). Although our tools are working as expected, we still observed partial conversion or Cre-mosaicism in our experiments. While injection of 300 pg of iCre mRNA or 1 ng of cell-permeant Cre protein led to satisfactory conversion, with 81% of all injected fish fully converted for the mRNA injections and 86% for the protein, we still observed some partially floxed animals (Figures 2D, E). 10pg of protein led to 100% conversion in all animals injected, but this comes with toxicity that may not be acceptable depending on the application. Similarly, with transgenic lines, while maternal deposition of iCre mRNA/protein led to 100% homogeneous conversion in all the conditions we tested, working with zygotic iCre expression still led to a few animals presenting some partial conversion and/or Cre-mosaic patterns. Also, very powerful and efficient, these observations should warn researchers to be cautious when working with tissue-specific lines and assume that full conversion is not always guaranteed in all the samples analysed.

Although more in-depth experiments and comparisons would be required, to improve this potential problem of partial conversion, we anticipate that controlling and limiting the number of Lox-responder insertions/copies would be strongly beneficial, as previously suggested in rodent studies (Heffner et al., 2012; Bao et al., 2013). This may be particularly important if the recombination efficiency cannot be controlled via the use of specific fluorescent markers as presented in this study.

With the increase in reliability of genome editing techniques such as CRISPR/Cas9, the utility of such conditional Cre/Lox technology has never been stronger (Okoli et al., 2022; Roy et al., 2022). There is no doubt that this technology will ease the study of housekeeping genes, such as genes involved in development, sex determination and reproduction which are difficult to investigate with traditional knockout approaches (Lin et al., 2013). Additionally, the use of such a conditional system may be interesting for aquaculture and for the expansion of transgenic fish production. Indeed, while infertility is a necessary adjunct to exploiting transgenic or mutant animals, the field needs to develop innovative, safe and robust techniques to successfully induce sterility (Golpour et al., 2016). For this, this system may provide new alternatives for triggering sterility or controlling sex determination.

## Materials and methods

### Zebrafish husbandry and lines

All zebrafish lines and embryos were maintained, and animal work was carried out in accordance to guidelines of the Animal Ethics Committee, University of Queensland, Australia. Ethics approval number AE213_18/AE213_18.

### Construction of iCre destination clones and storage of all plasmids

iCre sequence was previously described by Shimshek et al., 2002 (Shimshek et al., 2002). We incorporated its full sequence within a Gateway pDonor221, and generated the middle clone 469-pME-iCre. For further incorporating P2A-BFP, we used an In-Fusion cloning approach to amplify iCre (without Stop) from 469-pME-iCre as well as BFP from a custom of our clone supplemented with the P2A sequence. The sequences were incorporated into 237-pME-MCS from the tol2kit to generate the final 135_pME-iCRE_p2a_tagBFP. Destination clone 141-pT3TS-iCre was produced via gateway LR-reaction mixing 469-pME-iCre, 302-p3E-polyA and 02-p3T3TS_R1R3, as previously described (Giacomotto et al., 2015). 1455-pDEST-miniTol2_R4R2_MCS was built through multiple rounds of cloning available on request, with full sequence and annotation available at Addgene. Common tol2kit 394-pDestTol2pA2 clone (Kwan et al., 2007) has been modified to incorporate minitol2 (Urasaki et al., 2006) sequence fused with a I-SceI (Grabher et al., 2004) sequence, enabling either tol2-induced integration or meganuclease integration. We used both sequences to give the choice to the investigator, while minitol2 is widely used in the zebrafish community, the I-SceI sequence may be a preferred choice to promote single copy integration. We further replaced the AttR3 site with an AttR2 gateway-compatible sequence to remove the need for 3′ clone/p3E in the LR reactions (This dramatically improves the reaction success rate). We finally inserted a Multi Cloning Site (MCS) using directional cloning using SphI/ScaII sites. 1456-pDEST-miniTol2_R4R2_Cryst-eGFP and 1457-pDEST-miniTol2_R4R2_Cryst-cherry were further designed by inserting the Cryst_eGFP or Cryst_mCherry cassettes amplified by PCR from clones kindly provided by Professor Peter Currie. All clones have been sequenced, validated and made available at Addgene along with their map and sequencing data. 114-UBI:iCre_crystGFP has been generated using a Gateway LR reaction mixing p5E-UBI with 469-pME-iCre and 1456-pDEST-miniTol2_R4R2_Cryst-eGFP. Following all cloning procedures, the positive clones were identified using a PCR screening strategy and purified using Invitrogen Gel Extraction and PCR Purification Kit (#K220001) according to the manufacturer’s instructions. The Lox-Responder sensor “UBI:LoxSENSOR” clone presented in Figure 1B has been previously made by combining 101_p5e-Ubiquitin, 50_p5E-UBI-loxTagBFP and 010-pME-mKate with our destination clone 1457-pDEST-minTol2 R4-R2 Cryst-cherry (all available in addgene Table 1, or on request).

### mRNA synthesis and Cre-protein

iCre-mRNA was synthetised from 141-pT3TS-iCre using an mMessage mMachineTM T3 Transcription kit (Cat#AM1348). 141-pT3TS-iCre was first linearised by KpnI digestion. Following the T3 reaction, the mRNA was purified using a Zymo Research RNA Clean & Concentrator Kit (Cat#R1015) and eluted in 30 µL of Invitrogen Ultra-Pure Distilled water with a final concentration of ∼1 μg/μL aliquoted and stored at −80°C.

Cre protein or TAT-Cre Recombinase from Millipore (Cat# SCR508, Lot# 3015442) was suspended in Invitrogen Ultra-Pure Distilled water at a final concentration of 9.48 μg/μL. Aliquots of 2 µL were frozen at −20°C for long-term storage.

### mRNA and transgenes injections

For mRNA injections, we serially diluted RNA from a concentration of ∼1 μg/μL to concentrations of 300 ng/μL, 150 ng/μL, 100 ng/μL and 50 ng/μL with Invitrogen Ultra-Pure Distilled water. The injection mix was prepared on ice on the morning of injections such that the final volume to inject would be 1 nL into the yolk of the embryos as previously described (Tromp et al., 2021). For protein injections, Cre protein concentration was first adjusted to 5 μg/μL and then serially diluted to 1 μg/μL, 0.5 μg/μL, 0.1 μg/μL and 0.01 μg/μL with Invitrogen Ultra-Pure Distilled water. The injection mix was prepared on ice on the morning of injections such that the final volume to inject would be 2 nL into the cell of the embryos (Punnamoottil et al., 2015). All embryos after injections were collected and stored at 28°C in E3 medium. The next morning these were quantified as healthy, dead or malformed and subsequently imaged at 4dpf. For Cre DNA injections and selection of transgenics, we co-injected 1 nL of 25 ng/μL of Tol2 constructs (Figure 1) with 25 ng/μL of transposase RNA into one-cell embryos of wild-type AB strains. The Tol2 transposase RNA was generated using the Ambion mMessage mMachine T3 kit (Cat#AM1348). Injected embryos were screened at 2dpf for either GFP expression in their lens (Cre-Driver lines) or BFP expression within their body (Lox-Responders). Screened animals were grown up to adulthood and screened for F0 founders upon sexual maturation. F1 lines were then generated and used in this study.

### Toxicity testing

Embryos injected with mRNA and protein were analysed at 1dpf to report on the condition of the embryos post injections, as previously described (Restuadi et al., 2022). Embryos were categorized into healthy, dead and malformed. Malformations included obvious body defects such as oedema, stunted growth, blood pooling and other severe abnormalities with very slim chances to survive. These were observed in their Petri-dishes under a brightfield microscope. Numbers were recorded on an Excel spreadsheet and plotted using GraphPad Prism 9.0 software. Only healthy embryos were used for experiments.

### Imaging and quantification

The presence, absence, and relative intensity of BFP and mKate fluorescence were analysed using an Olympus MVX10 fluorescent microscope in dark condition. Quantification of dead, malformed and wildtype embryos post injections were manually counted under a compound light microscope. For image acquisition and in-depth analysis of fluorescence expression, animals were mounted laterally at 4dpf in 1% low-melting temperature agarose (Sigma-Aldrich, A9414) and imaged on a spinning-disk confocal system (Marianas; 3I, Inc.) consisting of an Axio Observer Z1 (Carl Zeiss) equipped with a CSU-W1 spinning-disk head (Yokogawa Corporation of America), ORCA-Flash 4.0 v2 sCMOS camera (Hamamatsu Photonics), with a 20×/0.8NA/550µm/PlanApo objective. Image acquisition was performed using SlideBook v6.0 (3I, Inc). Lasers 405 nm were used to record BFP fluorescence and 561 nm to record mKate fluorescence. All images exported as 16-bit TIFs were processed using Fiji software and exported as jpg for presentation. Data were plotted and compared using both Microsoft Excel and GraphPad Prism 9 software.

## Data Availability

The raw data supporting the conclusion of this article will be made available by the authors, without undue reservation.
